# Imaging the inflammatory phenotype in migraine

**DOI:** 10.1186/s10194-022-01430-y

**Published:** 2022-06-01

**Authors:** Rune Häckert Christensen, Cédric Gollion, Faisal Mohammad Amin, Michael A. Moskowitz, Nouchine Hadjikhani, Messoud Ashina

**Affiliations:** 1 Danish Headache Center, Department of Neurology, Rigshospitalet Glostrup, Faculty of Health and Medical Sciences, University of Copenhagen, Glostrup, Denmark; 2 Department of Neurology, University Hospital of Toulouse, Toulouse, France; 3 Department of Neurorehabilitation/Traumatic Brain Injury, Rigshospitalet, University of Copenhagen, Copenhagen, Denmark; 4 Massachusetts General Hospital, Harvard Medical School, Charlestown, MA USA; 5 Gillberg Neuropsychiatry Center, Sahlgrenska Academy, Gothenburg University, Gothenburg, Sweden

## Abstract

Several preclinical and clinical lines of evidence suggest a role of neuroinflammation in migraine. Neuroimaging offers the possibility to investigate and localize neuroinflammation *in vivo* in patients with migraine, and to characterize specific inflammatory constituents, such as vascular permeability, and macrophage or microglia activity. Despite all imaging data accumulated on neuroinflammation across the past three decades, an overview of the imaging evidence of neuroinflammation in migraine is still missing.

We conducted a systematic review in the Pubmed and Embase databases to evaluate existing imaging data on inflammation in migraine, and to identify gaps in the literature. We included 20 studies investigating migraine without aura (*N* = 4), migraine with aura (*N* = 8), both migraine with and without aura (*N* = 3), or hemiplegic migraine (*N* = 5).

In migraine without aura, macrophage activation was not evident. In migraine with aura, imaging evidence suggested microglial and parameningeal inflammatory activity. Increased vascular permeability was mostly found in hemiplegic migraine, and was atypical in migraine with and without aura. Based on the weight of existing and emerging data, we show that most studies have concentrated on demonstrating increased vascular permeability as a marker of neuroinflammation, with tools that may not have been optimal. In the future, novel, more sensitive techniques, as well as imaging tracers delineating specific inflammatory pathways may further bridge the gap between preclinical and clinical findings.

## Introduction

The hallmark of a migraine attack is severe headache accompanied by photophobia, phonophobia, and/or nausea [1], symptoms that are shared by other conditions characterized by meningeal inflammation. In animal models of migraine, neurogenic inflammation develops within the meninges and is mediated in part, by the release of vasoactive neuropeptides such as calcitonin gene-related peptide (CGRP), a molecule that plays a central role in migraine pathophysiology [2], and by cortical spreading depression, the mechanism strongly hypothesized to underlie aura [3]. This response is characterized by plasma extravasation, mast cell degranulation and possibly microglia/macrophage activation [3–8]. However, even though meningeal inflammation has not been consistently detected in clinical studies, more recently, different neuroimaging studies have supported the presence of an inflammatory signal in migraine patients. These have allowed *in vivo* visualization of inflammatory markers in human brain and surrounding tissues. Modalities and techniques include magnetic resonance imaging (MRI) to investigate macrophage-mediated inflammation [9] and extravasation [10, 11], single-photon emission computed tomography (SPECT) to evaluate extravasation [12], and positron emission tomography (PET) to assess activation of microglia and other inflammatory cell types [13, 14].

This article provides a systematic review of human neuroimaging studies focusing on inflammatory markers and their changes in patients with migraine. It critically appraises the weight of existing and emerging data, and evaluates the limitations of current methods used to study neuroinflammation. Finally, it considers how novel approaches, including those looking at inflammatory changes in the cortex and meninges, could play an important role in elucidating the involvement of neuroinflammation in migraine attacks, and potentially developing biomarkers for migraine.

## Methods

A systematic review was conducted. Articles were identified through the PubMed and Embase databases using the search algorithm: Migraine AND (Inflammation OR macrophages OR microglia OR permeability OR edema) AND (MRI OR Gadolinium OR PET OR Positron Emission Tomography OR SPECT OR Single Photon Emission Computed Tomography). These search terms were decided on since they are imaging techniques that allow visualizing inflammatory changes in the CNS. Databases were searched from inception until 22^nd^ of February 2022. In addition, articles were identified through references in the studies found by the search algorithms and by expert consultation. Two investigators (R.H.C. and C.G.) screened articles and extracted data.

Inclusion criteria were: imaging studies, clinical trials, case reports, or case series using imaging techniques considered sensitive to inflammatory changes (MRI with contrast agents, PET or SPECT techniques). Furthermore, only studies with migraine patients (including patients with debut of migraine or familial hemiplegic migraine) were included.

Exclusion criteria were: non-original articles, reviews, non-human studies, non-imaging techniques, non-migraine conditions, or studies providing no assessment of inflammatory changes. Studies examining e.g. cytotoxic edema without tracers were not included due to the limited specificity of this finding to inflammation.

## Results

**Fig. 1:**
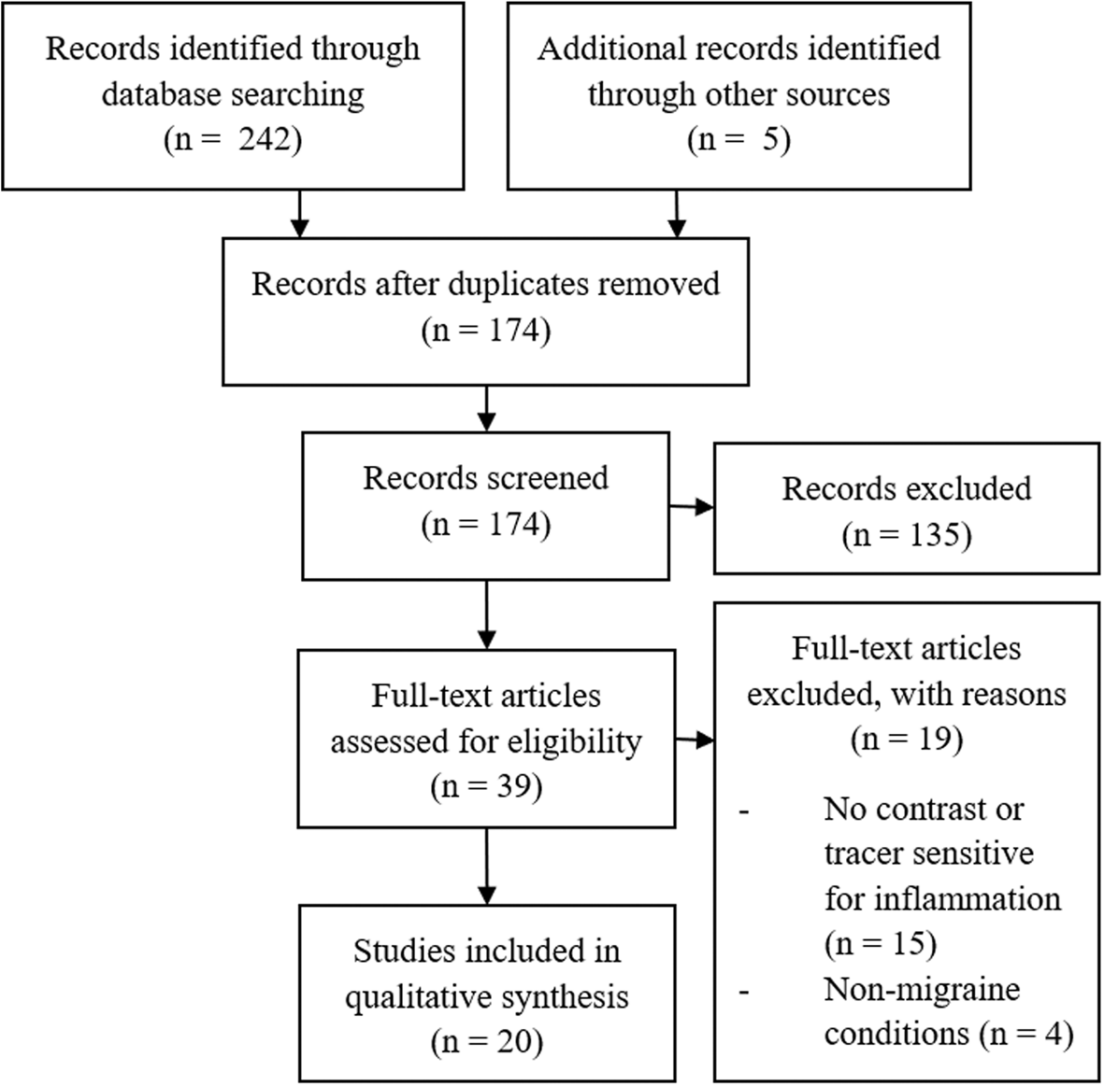
Preferred Reporting Items for Systematic Reviews and Meta Analyses (PRISMA) workflow chart of identified, excluded and included articles

We identified 169 unique records based on database search and five records from other sources. Exclusion of 135 records was based on abstract reading. Nineteen full-text articles were assessed and excluded as they did not include imaging parameters assessing inflammatory changes (either tracers examining inflammatory cell types or contrast agents examining extravasation). Twenty studies were included in qualitative analysis Fig. 1.

Of the 20 included studies, 4 investigated migraine without aura, 8 migraine with aura, 3 both migraine with and without aura, and 5 hemiplegic migraine. Fifteen studies used MRI contrast agents to examine inflammation, three used PET tracers, and 2 used SPECT tracers. For an overview of results from individual studies, see Tables 1, 2, and 3. In the tables, studies which included an evaluation of possibly meningeal enhancement or uptake are separated from studies which did not. Perfusion data are reported where this supports interpretation of the results. In the following, we present results from individual structured studies which carried out statistical analysis, and overviews of results from case reports or studies without statistical analysis within their respective sections.

**Table 1:** MRI contrast agent studies in migraine patients with (MA) and without aura (MO) to detect an inflammatory phenotype

**MO/MA studies which reported on dural enhancement**							
**Reference**	**Population (MA/MO)**	**Modality and contrast agent**	**Assumptions of technique**	**Delay after migraine onset**	**Comparisons**	**Results**	**Comments**
Khan et al. [9] 2019 Cephalalgia	MO w/ unilateral headache (cilostazol induced) *N* = 28	MRI w/ USPIO (ferumoxytol) Injected neat	USPIO binds to macrophages reflecting increased cell number and activation.	>24 h after headache onset	Ictal and post-ictal MO:	• No asymmetric uptake within brain parenchyma or ICA or MCA walls • No visual enhancement in MO or controls in brain parenchyma, vessels walls and dura • Uptake unaffected by ongoing headache • Uptake reduced by sumatriptan in ACA territory on pain-side (post-hoc analysis)	• USPIO tracer and sample size did not allow detection of minor changes in BBB permeability changes • Acute treatment of migraine attacks with sumatriptan may have abrogated inflammation • Scan conducted 27 h after contrast infusion
					Pain-side vs. non pain-side		
					Sumatriptantreated vs. w/o treatment		
Merli et al. [15] 2022 Headache	Mx *N* = 7 (5 MA, 2 MO) Episodic CH *N* = 8	Vessel-wall MRI w/ Gadolinium (unspecified)	Gadolinium passes a disrupted BBB	<24 h	Mx (ictal) vs. Mx (interictal)	• Focal linear enhancement in vertebral artery in one patient, present during and outside attack (attributable to an atheromatous plaque) • No vessels wall enhancement in intradural intracranial vessels during or outside attacks for remaining patients with migraine or cluster headache	• Case series • Acute treatment of migraine attacks (NSAID and triptans) may have abrogated inflammation • Diffuse vasoconstriction in two sumatriptan treated patients (one with migraine, one with cluster headache)
**MO/MA studies which did not report on dural enhancement**							
**Reference**	**Population (MA/MO)**	**Modality and contrast agent**	**Assumptions of technique**	**Delay after migraine onset**	**Comparisons**	**Results**	**Comments**
Kim et al. [16] 2019 Neuroradiology	Mx *N* = 35 (14 MO, 21 MA) HC *N* = 21	DCE MRI w/ Gadolinium (Gadobutrol)	Gadolinium passes a disrupted BBB	NA (interictal)	Mx (interictally) vs. HC	• Vascular permeability parameters similar for migraine patients and HCs	• MO and MA were not analyzed separately • Major variability in transfer constant for gadolinium compared to previous studies • Lower age of migraine patients as compared to control group (contrary to age related increases in BBB permeability) • Gadobutrol may be unable to extravasate during minor increases in BBB permeability
Amin et al. [11] 2017 European Journal of Neurology	MO (spontaneous) *N* = 19	DCE MRI w/ Gadolinium	Gadolinium passes a disrupted BBB	6.5 h (range 4.0-15.5 h)	MO (headache phase) vs MO (interictal) MO (pain-side vs. non-pain side)	• No increased permeability of gadolinium during attacks • No correlation between permeability and clinical features • No difference between early scan (less than 6.5h after attack onset) and late scan (more than 6.5h)	• Gadobutrol may be unable to extravasate during minor increases in BBB permeability • Permeability changes smaller than 35% could not be excluded • Mean of 28 days (range 12-87 days) between scans • No HC group
Hougaard et al. [10] 2017 Brain	MA with visual aura (spontaneous) *N* = 19	DCE MRI: Gadolinium (Gadobutrol)	Gadolinium passes a disrupted BBB	7.6 ± 5.8 h (time from aura onset)	MA (post-aura) vs. MA (interictal)	• BBB permeability was not different between post-aura and interictal scans, lateralized to one side, or different between patients experiencing scotomas with or without sharp edges • Increase in CBF for brainstem (bilateral), visual cortex (bilateral), and posterior cerebral hemisphere (symptomatic hemisphere)	• Gadobutrol may not be sensitive for minor increases in BBB permeability • Timing of scan may be unable to detect transient changes • Permeability differences smaller than 11% could not be excluded (post hoc analysis) • No HC group
Rotstein et al. [17] 2012 Cephalalgia	MA (spontaneous) *N* = 1	MRI w/ Gadolinium (Gd-DTPA)	Gadolinium passes a disrupted BBB	3 h after aura onset	MA (left hemisphere) vs. MA (right hemisphere)	• Unilateral (left sided) holohemispheric increase in BBB permeability during aura phase • Decreased CBF (hypoperfusion) in left hemisphere	• Case report • Left-sided headache with aphasia. • Prolonged aura (8 h)
Smith et al. [18] 2002 Neurology	MA (spontaneous) *N* = 1	MRI w/ Gadolinium (un-specified)	Gadolinium passes a disrupted BBB	48 h	MA (aura + headache phase) vs. MA (interictal) MA (symptomatic hemisphere) vs. MA (asymptomatic hemisphere)	• Vascular permeability increased in anterior temporal lobe of symptomatic hemisphere • MTT reduced in symptomatic hemisphere • Increased CBF (hyperperfusion) in symptomatic hemisphere	• Case report • Prolonged aura with hemiplegia in a patient with urinary tract infection, recent withdrawal of migraine preventive treatment (verapamil) and with an unremarkable lumbar puncture
Lanfranconi et al. [19] 2009 Journal of the Neurological Sciences	MA (spontaneous) *N* = 1	MRI w/ Gadolinium (un-specified)	Gadolinium passes a disrupted BBB	3h after relapse of aura	MA (aura and headache phase) vs. MA (interictal)	• Extravasation to CSF in left hemisphere	• Case report • 67-year old woman with debut of prolonged aura accompanied by aphasia, apraxia, and right-sided hemianopsia (right-handed patient)
Arnold et al. 1998 [20] Cephalalgia	MA/SHM (spontaneous sensorimotor aura) *N* = 1	MRI w/ Gadolinium (Gd-DTPA)	Gadolinium passes a disrupted BBB	One day after third attack	MA (interictal one day after attack) MA (interictal seven months later)	• Hyperintensity in left parieto-occipital white-matter on T2 and enhancement on T1 gadolinium	• Case report Lumbar puncture with slight lymphocytic pleocytosis (10 cells/mm^3^). No fever, elevated blood leukocytes, or elevated CRP • Only three episodes within an interval of 14 days
Gómez-Choco et al. 2008 [21] Neurology	MA (spontaneous sensorimotor aura) *N* = 1	MRI FLAIR w/ Gadolinium	Gadolinium passes a disrupted BBB	>10 h	MA (symptomatic hemisphere) vs. MA (asymptomatic hemisphere) MA (post-aura phase) vs. MA (interictal)	• Sulcal hyperintensity surrounding the left temporal lobe all the way up to the convexity during the post-aura phase on FLAIR 10 h after gadolinium infusion	• Case report • Sensory aura

^*11*^*C-DHE* 11-carbon dihydroergotamine, *BBB* blood-brain barrier, *CH* cluster headache, *CT* computed tomography, *DCE-MRI* dynamic-contrast enhanced magnetic resonance imaging, *Gd-DTPA* gadopentetic acid, *MA* migraine with aura, *MO* migraine without aura, *MTT* mean transit time, *Mx* migraine with or without aura unspecified, *PET* positron emission tomography, *SPECT* single-photon emission computed tomography, *USPIO* ultrasmall superparamagnetic iron oxides, *VAS* visual analogue scale (for pain)

**Table 2:** Imaging studies in familial and sporadic hemiplegic migraine (FHM/SHM) to detect an inflammatory phenotype

**FHM/SHM studies which reported on dural uptake**							
**Reference**	**Population (SHM/FHM)**	**Modality and contrast agent/tracer**	**Assumptions of technique**	**Delay after migraine onset**	**Comparisons**	**Results**	**Comments**
Dreier et al. [22] 2005 Neurology	FHM (ATP1A2 mutation carrier) (spontaneous) *N* = 1	MRI w/ Gadolinium (Gd-DTPA)	Gadolinium passes a disrupted BBB	1, 3, and 9 days after admission. Interictal scan after 17 months	FHM (aura phase) vs. FHM (interictal)	• Meningeal enhancement and BBB opening on the left hemisphere during aura phase • Pronounced contralateral cortical edema at day 9 on T2-weighted images	• Case report • Concomitant fever and neck stiffness • Lumbar puncture unremarkable • Neuropsychiatric deficits persisting for 3 months
**FHM/SHM studies which did not report on dural uptake**							
**Reference**	**Population (SHM/FHM)**	**Modality and contrast agent/tracer**	**Assumptions of technique**	**Delay after migraine onset**	**Comparisons**	**Results**	**Comments**
Cha et al. [23] 2007 Cephalalgia	FHM (spontaneous) *N* = 2	MRI w/ Gadolinium PET w/ 18-FDG	Gadolinium enhancement increases with perfusion and a disrupted BBB 18-FDG uptake increases with cerebral metabolism	During hemiplegia	Symptomatic hemisphere vs. Asymptomatic hemisphere Before hemiplegic episode vs. During hemiplegic episode	• Cortical occipital edema contralateral and to a lesser extend ipsilateral to headache, persisting 9 days into attack in one case • Gadolinium enhancement in right posterior gyri • 18-FDG uptake increased in contralateral temporal, insular, and occipital lobes	• Case reports • Twins • One case with reduced level of consciousness and followed by lasting neurological deficits • CSF with elevated protein, otherwise unremarkable • A right temporal lobe and dural biopsy showed reactive lymphocytes and astrogliosis • Uptake of 18-FDG increases with neuronal activity and is not specific for inflammation
Iizuka et al. [24] 2011 Journal of Neurology, Neurosurgery, and Psychiatry	FHM (spontaneous) *N* = 2 (6 attacks w/ contrast, 2 attacks w/o contrast)	MRI FLAIR w/ Gadolinium (unspecified) CBF-SPECT w/ HMPAO or IMP	Gadolinium passes a disrupted BBB HMPAO and IMP measures perfusion	Day 1-4 from aura onset	Ictal FHM (symptomatic hemisphere) vs. Ictal FHM (asymptomatic hemisphere)	• Early mild unilateral cortical edema at FLAIR (for 1/5 attacks in 1/2 patients) • Late cerebrospinal fluid enhancement on FLAIR in affected cortex in 1/3 attacks suggesting BBB leakage • Hyperperfusion in symptomatic hemisphere in 5 attacks and hypoperfusion in 3 attacks	• Gadolinium contrast may be unable to extravasate during minor decreases in BBB function • ATP1A2 mutation carriers • Early and late enhanced FLAIR was conducted 5-10 min and 2 post-contrast infusion, respectively
Iizuka et al. [25] 2006 Cephalalgia	SHM (spontaneous) *N* = 1	MRI (FLAIR w/ contrast): Gadolinium-based (Gd-DTPA)CBF-SPECT w/ HMPAO	Gadolinium passes a disrupted BBBHMPAO and IMP measures perfusion	Day 4	SHM (aura phase) vs. SHM (interictal)	• BBB permeability increased in left posterior cortex • Increased CBF (hyperperfusion) of left hemisphere • Reduced blood flow (hypoperfusion) of right cerebellum	• Case report • Concomitant aphasia, right-sided hemiplegia, confusion, and agitation (right-handed patient)
Pellerin et al. [26] 2019 Cephalalgia	SHM (spontaneous) *N* = 1	MRI contrast enhanced T1-spin echo weighted images, FLAIR, and DWI (contrast unspecified)	Gadolinium passes a disrupted BBB	NA (during aura)	Ictal FHM (symptomatic hemisphere) vs. Ictal FHM (asymptomatic hemisphere) FHM (ictal) vs. Ictal FHM (interictal)	• Diffuse cortical enhancement of right hemisphere on T1-spin echo weighted images • Slight hyperintensity on FLAIR • Slight hyperintensity on DWI • Hyperperfusion of right hemisphere on non-contrast ASL	• Case report • Left-sided hemiplegia with headache and altered consciousness • Right frontal lobe biopsy with advanced neuronal suffering, ballooned cells, neoangiogenesis and fibrohyalinosis

*18-FDG* 18-fluorodeoxyglucose, *ATP1A2* Na^+^/K^+^ transporting ATPase subunit alpha-2, *BBB* blood-brain barrier, *CT* computed tomography, *DWI* diffusion-weighted imaging, *FHM* familial hemiplegic migraine, *Gd-DTPA* gadopentetic acid, *HMPAO* 99mTc-D,L-hexamethyl-propyleneamine oxime, *IMP* N-isopropyl-p-^123^I iodoamphetamine, *PET* positron emission tomography, *SHM* sporadic hemiplegic migraine, *SPECT* single-photon emission computed tomograph

**Table 3:** PET/SPECT studies in migraine patients with (MA) and without (MO) aura to detect an inflammatory phenotype

**MO/MA studies which reported on dural uptake**							
**Reference**	**Population (MA/MO)**	**Modality and Tracer**	**Assumptions of Technique**	**Delay after Migraine Onset**	**Comparisons**	**Results**	**Comments**
Hadjikhani et al. [14] 2020 Annals of neurology	MA with visual aura *N* = 11 HC *N* = 11 Chronic lower back pain *N* = 11	PET/MRI w/ ^11^C-PBR28	^11^C-PBR28 ligand binds to mitochondrial receptor protein on many activated cell types during inflammation.	8 days median (0-18 range) 1 patient ictal during scan	MA with visual aura (interictally) vs. HC vs. Chronic lower back pain	• Uptake increased in MA in parameningeal tissue (meninges and skull bone) overlying occipital cortex • Uptake correlated with total episodes of visual aura in the preceding 4 weeks • Uptake unrelated to total attacks in the preceding 4 weeks	• ^11^C-PBR28 does not distinguish among cell types activated during inflammation • Total attack duration and intensity was not considered
Knotkova et al. [12] 2007 Pain Medicine and Pappagallo et al. [27] 1999 Neurology (*Preliminary* results)	MO (spontaneous) *N* = 2	SPECT w/ 99-Tc human serum albumin	99-Tc albumin to detect disrupted BBB	1^st^ scan: 3 and 12 h after migraine onset 2^nd^ scan: 3 h after 1^st^ scan 3^rd^ scan: Interictal	MO (ictal) vs. MO (interictal)	• Increased extravasation on the second scan at 3 h in frontotemporal and frontal regions ipsilateral to headache	• Preliminary results • Case reports
**MO/MA studies which did not report on dural uptake**							
**Reference**	**Population (MA/MO)**	**Modality and tracer**	**Assumptions of technique**	**Delay after migraine onset**	**Comparisons**	**Results**	**Comments**
Albrecht et al. [13] 2019 Neurology	MA *N* = 13 HC *N* = 16	PET/MRI w/ ^11^C-PBR28	^11^C-PBR28 ligand binds to mitochondrial receptor protein on many activated cell types during inflammation, including microglia.	8.08 ± 5.03 days (mean ± SD)	MA (interictally) vs. HC	• Uptake increased in visual cortex, thalamus, primary and secondary somatosensory cortices, posterior insular cortex, primary motor cortex, auditory cortices, regions of prefrontal cortex, orbitofrontal cortex, putamen, area MT and V3A • Uptake correlated with frequency of migraine attacks in several cortical and subcortical areas	• ^11^C-PBR28 does not differentiate between cell types activated during inflammation
Schankin et al. [28] 2016 Brain	Mx *N* = 6 (4 MO, 2 MA) (GTN induced migraine attacks without aura) HC *N* = 6	PET/CT w/ ^11^C-DHE	^11^C-DHE to detect DHE passage of the BBB	Baseline scan and scan at 3 h after GTN infusion	Mx (Ictal) vs. Mx (interictal) HC (baseline) vs. HC (post-GTN)	• For Mx, no difference between ictal and interictal uptake • For HCs, no difference between baseline and post-GTN uptake • For all participants, uptake in choroid plexus of the lateral and 4^th^ ventricles, pituitary fossa, venous sinus and facial tissue, and no uptake in cortical areas, brainstem, or thalamus	• Whether DHE passes a disrupted BBB is unexamined • Sample size may not have allowed detection of minor changes in BBB permeability
Sianard-Gainko et al. [29] 1993 Cephalalgia	MO *N* = 7 Cluster headache *N* = 30	SPECT w/ Gallium 67 citrate	Gallium 67 binds to proteins in inflammatory exudates [30].	NA	MO (interictal)Cluster headache (interictal, chronic and episodic in active period)	• High parasellar uptake in 5/7 MO patients • High parasellar uptake similar for MO and cluster headache	• Absence of quantitative data may have overestimated uptake • No HC group

^*11*^*C-DHE* 11-carbon-dihydroergotamine, ^*11*^*C-PBR28* [*O-methyl*-^11^C]-*N*-acetyl-*N*-(2-methoxybenzyl)-2-phenoxy-5-pyridinamine; ^*99*^*Tc* 99-technetium, *BBB* blood-brain barrier, *CT* computed tomography, *GTN* glyceryl trinitrate, *HC* healthy control, *MA* migraine with aura, *MO* migraine without aura, *Mx* migraine with and without aura, *NA* not applicable, *PET* positron emission tomography, *SPECT* single-photon emission computerized tomography

### MRI with contrast agents

A key feature of inflammation is vascular permeability. Because gadolinium-based MRI contrast agents extravasate through leaky vessels, gadolinium enhancement and transfer rates provide estimates of vascular permeability (Table 4).

**Table 4:** Assumptions of gadolinium-contrast MRI

Gadolinium decreases T1 relaxation time which appears as increased intensity on T1-weighted images. The concentration of gadolinium in a tissue is proportional to the T1 signal intensity. By scanning before and after gadolinium infusion, the amount of gadolinium which was extravasated can be qualitatively estimated by increased T1 intensity, i.e. enhancement. DCE-MRI provides a quantitative measurement of BBB leakage. In DCE-MRI, an image is acquired pre-contrast infusion and then multiple images are acquired during contrast infusion. The multiple T1 values are used to calculate the rate at which T1 signal intensity increases during infusion. This provides a quantitative measure of how quickly gadolinium extravasates, i.e. the permeability, named the volume transfer constant (K_trans_).	

#### Migraine without aura

Three structured studies, one case report, and one uncompleted study investigated the presence of increased vascular permeability (disruption of the blood-brain barrier (BBB)) in migraine without aura (MO) (Table 1). All but one MRI study in patients with MO used gadolinium or derivatives thereof (see Table 1). Only one study analyzed BBB permeability in the interictal period in 14 patients with MO and 21 patients with migraine with aura (MA) [16]. All other studies were performed in the ictal period.

Amin et al. examined 19 patients with MO during and outside of attacks using gadolinium contrast [11]. Patients were scanned a mean of 6.5 h after attack onset. The study found no change in BBB permeability for gadolinium between the ictal and interictal scan for any of the regions of interest (ROIs) (including hemispheres, anterior, middle, or posterior cerebral areas, brain stem areas, or posterior pons). For patients with unilateral head-pain during the attack, there were no differences between the pain side compared to the non-pain side for any of the ROIs.

Khan et al. examined macrophage activation in patients with MO using the MRI contrast agent ultra-small superparamagnetic iron oxide (USPIO), which is engulfed by activated macrophages and extravasates when the BBB is disrupted [9] (Table 1). Twenty-eight patients with MO ingested cilostazol and developed migraine attacks with unilateral headache, 12 of which were treated with sumatriptan subcutaneously. All 28 participants then received infusion with USPIO and were scanned 27 hours after infusion of USPIO. The 27 hour time point was selected since delayed USPIO uptake is thought to reflect cellular uptake, while USPIO also acted as a blood pool agent initially [31, 32]. The study found no difference in USPIO uptake for the pain side compared to the non-pain side in brain parenchyma, the middle cerebral artery, cavernous part of the internal carotid artery, or upon visual inspection of the dura mater. However, in post-hoc analysis, the transverse relaxation rate (ΔR2*) was increased bilaterally in the anterior cerebral artery territory for the group without sumatriptan treatment, and ΔR2* was higher on the pain-side for the untreated patients. Tissue uptake of USPIO increases ΔR2* [33].

One cases series comprising seven patients with migraine (5 MO, 2 MA) investigated intradural intracranial vessel wall enhancement with gadolinium, but found no enhancement during or outside of attacks in 6 patients. The remaining patient had focal vertebral artery enhancement, but this persisted interictally and was likely attributable to an atherosclerotic plaque [15], which gadolinium contrast enhances [34]. An unclear number of the patients had consumed anti-inflammatory analgesics or triptans.

#### Migraine with aura

Three structured studies and five case reports have examined the presence of increased vascular permeability in MA (Table 1).

Three case reports showed meningeal enhancement in gadolinium-contrasted MR that evocated BBB leakage, during prolonged or atypical auras in MA [18–20]. One other case report found holohemispheric enhancement [17], and another sulcal hyperintensity on gadolinium enhanced FLAIR [21].

Hougaard et al. examined BBB permeability for gadolinium in 21 patients with MA after aura compared to attack-free days [10]. The mean time from aura onset until scan was 7.6 h. The study found no differences in BBB permeability after aura compared to attack free days, and no difference between hemispheres. There was no correlation between BBB permeability and the time from symptom onset until scan. No healthy controls (HCs) were included for comparison. Kim et al. compared BBB permeability in 35 interictal patients with migraine (21 patients with MA, and 14 with MO) with 21 HCs using gadolinium contrast [16]. The study found no differences in BBB permeability between the groups.

#### Hemiplegic migraine

In hemiplegic migraine, one case series reported eight attacks in two patients of the same family. Six of the eight attacks were analyzed with contrast agent and only one attack showed cortical enhancement in the symptomatic brain area [24].

Four case reports have observed gadolinium-contrast MR enhancement of the meninges which could be accompanied by cortical edema [22, 23, 25, 26]. These findings revealed permeability changes, that repeatedly occurred in the gyri, but were also present in the dura matter (Table 2) [22].

### PET/SPECT

In PET and SPECT technique, radioactive tracers are used to locate and quantify specific molecules to determine their possible pathophysiological involvement (Table 5).

**Table 5:** Assumptions of PET/SPECT imaging

Radioactive tracers are composed of radioactive nucleotides linked to different ligands. The nucleotides emit positrons (PET) or photons (SPECT) from the tracer, which allows determining the tracer’s location. When the ligand is a hydrophilic plasma protein such as albumin, the tracer provides a measure of extravasation just like most MRI contrast agents. When the ligand binds specific molecular targets, such as TSPO, they can be used to elucidate specific pathophysiological processes.	

#### Migraine without aura

One case report and preliminary results from an incomplete study used technetium-99m labeled human serum albumin (99Tc-HSA) to estimate extravasation in patients with MO (Table 3). For one patient, there was SPECT enhancement along the right frontal convexity 3 hours after tracer infusion in an attack of migraine without aura [12, 27]. This corresponded to the location of the patient’s headache [12].

Another SPECT study examined parasellar uptake of gallium-67 citrate in migraine without aura, in the context of a study on cluster headache. The study included 7 patients with MO and found parasellar hyperactivity in 56% of patients with migraine. However, parasellar hyperactivity was also observed for cluster headache patients and no statistical comparison was made [29].

Schankin et al. examined BBB permeability with 11C-dihydroergotamine (^11^C-DHE) PET in glyceryl trinitrate (GTN)-induced migraine attacks without aura [28]. The study included 6 patients with migraine (4 with MO patients, 2 with MA) and 6 healthy controls. The study found no differences in uptake of ^11^C-DHE in patients with migraine during attacks compared to outside of attacks, or in healthy controls before GTN infusion compared to after GTN infusion. None of the healthy controls developed headache after GTN infusion in this study.

### Migraine with aura

The PET tracer ^11^C-PBR28 targets the membrane protein translocate protein (TSPO), which upregulates on multiple cell types, including microglia, macrophages, and astrocytes during inflammation [35, 36].

In patients with MA, a PET-MRI study used the tracer to detect inflammatory upregulation in the cerebrum of patients with MA interictally [13]. Thirteen patients with MA were compared to 16 healthy controls. All patients with MA had at least one aura attack within 15 days preceding the scan. Compared to healthy controls, patients with MA had widespread increased tracer uptake across several cortical sites, including occipital striate and extrastriate visual cortex, somatosensory cortex, insula, thalamus, and in the spinal trigeminal nucleus (Table 3). This was correlated with the number of migraine attacks per month in several cortical and subcortical areas including frontoinsular cortex, primary/secondary somatosensory cortices, and basal ganglia.

Using the same PET tracer, the group also examined uptake in parameningeal tissues (the dura mater, brain, and bone barrow) [14]. The study included 11 patients with frequent visual MA, who were scanned within 18 days after their last migraine attack with or without aura, and who had at least one aura attack within the preceding four weeks. Some of these patients also experienced MO. In addition, the study included control groups of 11 healthy controls and 11 patients with chronic lower-back pain. The study found increased uptake in the parameningeal tissue overlying the occipital cortex, when comparing to healthy controls and patients with chronic lower-back pain. In the occipital parameningeal tissue, the uptake was correlated with the total number of visual auras.

## Discussion

Our discussion is intended to examine the overlapping and complementary preclinical and clinical evidence to support human imaging findings in patients with MO and patients with MA. The strength and weakness of the data will be considered. When appropriate, recommendations will be made for how to proceed to advance the translational evidence from animal to human.

### Migraine without aura

#### Evidence of macrophage involvement

Preclinical findings suggest that macrophages participate in a delayed inflammatory response that may take place in the meninges in migraine [7, 37]. This was investigated using the nitric oxide-donor glyceryl trinitrate (GTN), which induced migraine attacks in 70-80% of patients with migraine when given intravenously [38]. When given to rodents, GTN activated dural macrophages along the middle meningeal artery to express proinflammatory and nociceptive inducible nitric oxide synthase (iNOS), interleukin-1β (IL-1β), and interleukin-6 (IL-6) [7]. The macrophages activated with a delay of 2 hours, comparable to the timing of migraine headache induced after GTN infusion [7, 39]. As histological examination is not feasible in humans, clinical studies examined monocytes in blood taken from the jugular vein during spontaneous migraine attacks instead, finding increased iNOS expression and higher levels of IL-1β and IL-6 [40, 41].

To localize a site for this monocyte/macrophage activation in patients with MO, an imaging study subsequently used the MRI contrast agent USPIO, which is engulfed by activated macrophages [9]. The study looked for lateralized differences in patients who experienced unilateral migraine headache after cilostazol ingestion. However, the study found no differences in side-to-side signal intensity in the middle cerebral artery (MCA) or cavernous segment of the internal carotid artery (ICA_cavernous_), for vascular territories supplied by the anterior cerebral artery (ACA), MCA, or posterior cerebral artery (PCA), for the pons or thalamus, or upon visual inspection of the brain parenchyma or dura mater. While patients who did not receive sumatriptan treatment for the attack had a higher uptake than patients who received treatment, this was investigated as a post-hoc analysis. It is possible that sumatriptan treatment could have attenuated macrophage activity in the primary analysis.

Of note, since the study did not compare the uptake of USPIO between patients with MO and HC, nor between during and outside an attack, no inferences can be made about changes in macrophage activity during attacks. To analyze attack-specific macrophage activity, uptake could be compared between patients who did and did not experience a migraine attack after receiving a migraine provoking substance, or between spontaneous migraine attacks and the interictal state. Furthermore, the timing of the study with scans 27 hours after transfer infusion, may not have permitted detection of transient changes in the beginning of the attack. Finally, statistical testing was not possible for the dura mater or the middle meningeal artery, where macrophage activation was initially implicated in preclinical studies.

#### Evidence of vascular permeability

Most imaging studies in MO have examined vascular permeability that could be the consequence of inflammation. In rats, antidromic trigeminal activation leads to release from C and Aδ fibers of inflammatory neuropeptides which induces dural plasma protein extravasation [42]. Neuropeptides such as substance P directly induces extravasation [42, 43], while others, e.g. CGRP, do so indirectly [44–46] by stimulating mast cell degranulation and histamine release [47, 48]. GTN infusion in rats caused a similar, but delayed plasma protein extravasation, suggesting an identical response could occur in patients with migraine [7]. Vascular permeability and BBB function could also deteriorate due to the activity of matrix metalloproteinases (MMPs), extracellular proteolytic enzymes that regulate inflammation and disrupt the BBB [49, 50]. While early findings suggested MMPs to be dysregulated in patients with MO [51–53], findings of increased MMP-9 levels during MO attacks [53, 54] could not be replicated in a study measuring in blood from the external jugular vein [55].

Neuroimaging offers the opportunity to visualize this extravasation in patients with MO. Two initial clinical cases reported an increased uptake in meningeal 99Tc-HSA ipsilateral to the patient’s migraine headache. The uptake was increased three hours after tracer injection, and not on early acquisition [12, 27], which suggests that the uptake corresponded to extravasation of the tracer and not only hyperemia [12]. However, how vascular changes evolve over time during migraine has not specifically been evaluated. Another study measured high parasellar uptake of gallium [29], whose uptake is enhanced by increased blood flow and vascular membrane permeability [56]. However, the uptake was also high in cluster headache, and the region is not relevant for migraine pathophysiology. To verify whether plasma protein extravasated during migraine attacks, structured human imaging studies subsequently used the transfer rate of gadolinium or hydrophilic molecules of a similar size to investigate and quantify BBB permeability [11, 16, 28]. However, scans of 25 MO patients during attacks (19 spontaneous, 6 GTN-provoked) found no difference in BBB permeability for gadolinium or the migraine treatment dihydroergotamine compared to the interictal state [11, 28]. Another study found no difference between patients with MO interictally and healthy controls, but substantial variation in the gadolinium transfer rate limited interpretation of this finding [16].

Importantly, the structured studies lacked the statistical power to detect minor differences in blood-to-brain leakage of gadolinium [11, 16], which may not be sufficiently sensitive to detect minor changes in vascular permeability. Furthermore, the structured studies could not directly examine the meninges, since the resolution of standard MRI is too low to explore the meninges specifically. However, a recent case series examined intradural vascular gadolinium uptake using vessel wall MRI during and outside attacks of migraine (mixed MO and MA). The time from headache onset until scan was not reported but was <24 hours for all patients [15]. The study did not find vessel wall enhancement in intradural intracranial vessels, but all participants had consumed anti-inflammatory analgesics or triptans before their scan, and therefore one cannot exclude that they may have quenched an inflammatory signal. Finally, no statistical comparison was performed, and the case series did not include healthy controls.

### Migraine with aura

Animal models of migraine with aura suggest a proinflammatory role of cortical spreading depression (CSD), the neural correlate of aura symptoms. In neurons, CSD activates a distinct inflammatory pathway by increasing Pannexin 1 megachannel opening and caspase-1 activation, subsequently resulting in neural high mobility group protein (HMGB1) release. This stimulates nuclear factor-κβ (NF-κβ) activation in astrocytes, inducing transcription of cytokines and proinflammatory enzymes and headache behavior in animal experiments [57].

#### Evidence of macrophage involvement

In rodents, manipulating the cortex with electrodes triggered CSD, which activated meningeal macrophages [58, 59]. In the dura, macrophage activation was delayed by 20 minutes, a time frame similar to the delay from aura onset until headache onset in the majority of patients with MA [60]. Macrophages may release cytokines such as IL-1β and tumor necrotizing factor-α (TNF-α) that are reported as elevated after CSD [61] and in patients with MA [62]. These could sensitize meningeal afferents directly (e.g. IL-1 [63] and TNF-α [64]) or indirectly by increasing CGRP release [65].

Though no imaging studies directly examined macrophage activation in patients with MA, one study reported increased uptake of the tracer ^11^C-PBR28 in patients with multiple attacks of migraine with visual aura within the parameningeal tissues (Table 3). TSPO is the peripheral benzodiazepine receptor and upregulates during inflammation in several cell types, including macrophages. Enhanced uptake was associated with the total number of visual auras in the preceding 4 weeks [14]. In these patients, averaging 8 attacks over the prior 30 days, enhanced tracer uptake persisted for at least 18 days after the last attack. It is unknown as yet whether this uptake occurs in migraine without aura [14].

Complementary to the above, a recent study using micro-computed tomography (μCT) demonstrated the existence of microvascular channels in human skull. These channels allow leucocytes derived from skull bone marrow to migrate towards the brain to reach the meninges [66], as demonstrated in chemical meningitis and stroke. To explain the enhanced ^11^C-PBR28 uptake in the bone marrow, it has been posited that inflammatory signal(s) generated in cortex following CSDs (e.g., cytokines and/or HMGB-1) are released and taken up by microvascular channels to reach the bone marrow. Within the bone marrow, these signals provoke the migration of myeloid cells towards the meninges overlying the occipital cortex, the source of CSD in visual auras. Indeed, the demonstration of enhanced tracer uptake in visual cortex overlying meninges, and adjacent bone marrow supports the above formulation after multiple migraine with aura attacks [14]. Whether the findings and formulation relate to the pathogenesis of recurrent and frequent migraine with aura attacks remain to be determined.

#### Evidence of microglia involvement

Animal models of migraine with aura suggest neuroinflammatory activation of microglia. As delineated above, cortical spreading depression initiates a complex cascade involving pannexin 1 channel opening with subsequent microglial activation. Like macrophages, activated microglia may release proinflammatory cytokines [67, 68] which can sensitize perivascular nociceptive afferents. In addition, activated microglia express TSPO [6, 69], which exhibited sustained upregulation in animals after CSD, when examined with a PET tracer [6].

Similar pro-inflammatory upregulation was replicated in patients with MA interictally with the TSPO tracer ^11^C-PBR28 [69], suggesting pro-inflammatory microglial activation or recruitment [70]. TSPO was upregulated in several regions previously implicated in MA, including occipital striate and extrastriate visual cortex, somatosensory cortex, insula, thalamus, and in the spinal trigeminal nucleus [13]. There was an association between frequent migraine attacks and uptake of the tracer in the human neuroimaging study, which may reflect a cumulative impact of multiple CSDs on microglial activation or number, sufficient to detect clinically. Similar cumulative effects of multiple CSDs have been observed preclinically [71].

Of note, ^11^C-PBR28 binds non-specifically to activated macrophages, monocytes, neutrophils, dendritic cells, mast cells, and adipocytes besides microglia [14]. Although this shortcoming limits the ability to dissect the contribution of individual cell types in the inflammatory process, it does have the advantage of detecting a composite of the inflammatory process in multiple cell types, thereby enhancing the overall signal intensity. The development of more cell type-specific ligand will add another dimension to these new and important findings.

#### Evidence of vascular permeability

In animal models of migraine, CSD induces extravasation of plasma proteins from dura mater vasculature, by activating trigeminal afferents [3, 57, 72]. In rats, trigeminal activation after CSD may activate and upregulate the BBB degrading enzyme MMP-9, which results in plasma protein leakage [49]. Though attacks in patients with MA have been associated with increased MMP-9 concentrations [53], it is uncertain whether MMP-9 levels differ between patients with MA interictally and healthy controls [51, 54].

In MA, a BBB breakdown has been visualized in a few clinical cases with contrast extravasation on MRI [17–21]. However, these were prolonged auras [17, 21] or atypical cases in which differential diagnoses such as hemiplegic migraine, cerebral amyloid angiopathy or transient headache and neurological deficits with cerebrospinal fluid lymphocytosis (HANDL) syndrome cannot be completely excluded [18–20]. In a structured study of typical cases of MA in 19 patients, Hougaard et al. found no increase in BBB permeability for gadolinium during the migraine headache [10]. However, comparisons to healthy controls were not made, and there was a lack of statistical power to detect minor permeability changes. In this study, patients were studied during hypoperfusion or hyperperfusion phases. Interestingly, in the case published by Rostein et al., the observed increase in BBB permeability observed was contemporaneous with ipsilateral hypoperfusion, which would suggest a BBB disruption unrelated to hyperemia [17]. To our knowledge, this is a unique observation.

#### Hemiplegic migraine

Hemiplegic migraines (HM) could be sporadic (SHM) or familial (FHM). Familial HM can be due to several genetic mutations, which all increase the susceptibility to CSD. In patients with HM, CSD could induce more pronounced extravasation of plasma proteins than in classical aura, since preclinical studies suggest multiple CSDs amplify plasma protein extravasation [71]. This corresponds to the severe neurological paresis characterizing the disorder.

One structured imaging study (*N* = 2) [24] and 4 case reports examined changes in vascular permeability in FHM, while one case report examined changes in vascular permeability in SHM (Table 2). Contrast agent extravasated in 5 out of 13 hemiplegic migraine attacks. These were always prolonged auras, and the extravasation of contrast agent was extensive, sometimes associated with cortical oedema, and was seen in a phase of hyperemia [22–26].

The increased vascular permeability reported in human neuroimaging studies of FHM [22–24] (Table 2) supports preclinical findings of leaky vessels after CSD [72]. However, there is a need for structured studies using sensitive methods to detect extravasation, to confirm these findings in a broader population of patients with FHM. Studying specific components of CNS inflammation, such as macrophage or microglial activation, as has been done in patients with MO and MA, would also be essential to determine if specific inflammatory pathways are involved in FHM.

### Future perspectives

Most human imaging studies have focused on changes in vascular permeability to detect a key feature of inflammation in brain and surrounding tissues. In MA, neuroimaging studies have found neuroinflammatory activity in the cortex (possibly microglial in origin) and parameningeal tissues (possibly monocytic in nature). Imaging signs of neuroinflammation in MO have been less convincing. Major disruption of the BBB has not been observed consistently, although subtle or transient changes cannot be ruled out. The presence of a BBB breakdown is mainly seen in cases of atypical and prolonged auras as in hemiplegic migraine. In the classic forms of aura this observation is exceptional, and in MO it is uncertain. Furthermore, it is not clear whether the observed barrier breaks are due to hyperemia or inflammation or both. Only one observation showing extravasation at a hypoperfusion phase supports the second hypothesis [17]. It is possible that the common methods used lack the sensitivity to detect subtle disruptions of the BBB [10]. Future studies should apply more sensitive methods to detect extravasation, e.g. dynamic contrast-enhanced MRI (DCE-MRI) with longer acquisition times, or methods other than those based on gadolinium tracer, e.g. detecting molecular diffusion of intra- and extra-cellular water with T1 and T2 mapping.

Novel tracers targeting molecules upregulated during specific inflammatory processes, such as the leukocyte trafficking molecule urokinase plasminogen activated receptor, could then define components of an inflammatory response further. However, there are still many relevant targets in migraine for whom tracers have not been developed yet.

Other methodological considerations apply to the study of neuroinflammation in migraine in general. Timing of scans with regard to migraine phase may be essential to detect transient changes; serial scans during the migraine prodrome, headache, and resolution should be conducted to determine the time window of such transient changes. Importantly, temporal considerations may depend on the individual inflammatory event. For example, preclinical and clinical studies suggest extravasation is most likely to occur a few hours into migraine attacks, whereas microglial activation may not begin until a few days after CSD [6]. Furthermore, studies should be sufficiently powered to detect discrete inflammatory changes in macrophage activity or BBB permeability.

Meningeal and vascular inflammation is relatively unexamined. Preclinical studies suggest this location to be a prime candidate for inflammation in MO [7]. The dura lacks a BBB and has a composition and resident inflammatory cells distinct from those in the brain. Imaging data show that the middle meningeal artery (MMA) dilates specifically on the pain side in cilostazol-induced attacks [73]. However, several studies with MRI contrast agents did not report whether there was an overt dural enhancement or not, but this would probably have been mentioned if present. Future studies should explicitly report whether dural enhancement was observed or not. With future high-resolution imaging techniques, it may be possible to analyze the meninges separately. Neuroimaging studies should also continue to explore inflammatory changes in the cortex of patients with MA, particularly in relation to CSD.

Establishing the role of inflammatory pathways in migraine pathophysiology could help identify locations and targets for specific anti-migraine treatments and contrast the different migraine types. For example, the extent to which the BBB is altered or not in migraine is essential to determine the site of action of current and future migraine treatments. Neuroinflammatory imaging signals may also become possible future biomarkers for migraine. Therefore, it will be critical to determine whether microglial activation occurs in MO (especially whether the tracer 11C-PBR28 shows similarities in MO and MA), if macrophage activation occurs in MA, and if either occurs in FHM.

In summary, brain imaging circa 2022 provides an essential tool to understand the natural history of migraine and is a viable way to reconcile emerging preclinical and clinical data. It also holds great promise for discovering and interrogating key biological processes in human brain underlying this enigmatic neurovascular disorder. At this point, more studies are needed along with more specific and selective markers of cells and tissues as well as efforts to harmonize the protocols and newly acquired data sets between laboratories.

## Acknowledgments

Dr. Gollion reports personal fees for consultancy from Teva. Dr. Amin has received honoraria and personal fees from Teva, Lundbeck, Novartis, Eli Lilly for lecturing or participating in advisory boards. Dr. Hadjikhani received grant support from the National Institute of Healthy, grant NIH-NCCAM 5P01AT009965-03. Dr. Ashina has received personal fees from AbbVie/Allergan, Amgen, Eli Lilly, Lundbeck, Novartis and Teva Pharmaceuticals, and is the principal investigator of ongoing clinical trials for AbbVie/Allergan, Amgen, and Lundbeck. He has received research grants from the Lundbeck Foundation, Novo Nordisk Foundation, and Novartis. He is associate editor of Brain, Cephalalgia, and The Journal of Headache and Pain. He is past President of the International Headache Society. Dr. Moskowitz and Dr. Christensen have no disclosures to report.

## References

[1] IHS (2018). The International Classification of Headache Disorders, 3rd edition (beta version), Headache Classification Committee of the International Headache Society. Cephalalgia.

[2] Moskowitz MA (1993). Neurogenic inflammation in the pathophysiology and treatment of migraine. Neurology.

[3] Bolay H, Reuter U, Dunn AK (2002). Intrinsic brain activity triggers trigeminal meningeal afferents in a migraine model. Nat Med.

[4] Pusic KM, Pusic AD, Kemme J, Kraig RP (2014). Spreading Depression Requires Microglia and is Decreased by their M2a Polarization from Environmental Enrichment. Glia.

[5] Magni G, Boccazzi M, Bodini A (2019). Basal astrocyte and microglia activation in the central nervous system of Familial Hemiplegic Migraine Type I mice. Cephalalgia.

[6] Cui Y, Takashima T, Takashima-Hirano M (2009). 11C-PK11195 PET for the in vivo evaluation of neuroinflammation in the rat brain after cortical spreading depression. J. Nucl. Med..

[7] Reuter U (2001). Delayed inflammation in rat meninges: implications for migraine pathophysiology. Brain.

[8] Huang Z, Byun B, Matsubara T, Moskowitz MA (1993). Time-dependent blockade of neurogenic plasma extravasation in dura mater by 5-HT1B/D agonists and endopeptidase 24.11. Br J Pharmacol.

[9] Khan S, Amin FM, Fliedner FP (2019). Investigating macrophage-mediated inflammation in migraine using ultrasmall superparamagnetic iron oxide-enhanced 3T magnetic resonance imaging. Cephalalgia.

[10] Hougaard A, Amin FM, Christensen CE et al (2017) Increased brainstem perfusion, but no blood-brain barrier disruption, during attacks of migraine with aura. Brain:1–10 10.1093/brain/awx089 28430860

[11] Amin FM, Hougaard A, Cramer SP (2017). Intact blood-brain barrier during spontaneous attacks of migraine without aura: a 3T DCE-MRI study. Eur J Neurol.

[12] Knotkova H, Pappagallo M (2007). Imaging intracranial plasma extravasation in a migraine patient: a case report. Pain Med.

[13] Albrecht DS, Mainero C, Ichijo E et al (2019) Imaging of neuroinflammation in migraine with aura: A [11C]PBR28 PET/MRI study. Neurology. 10.1212/WNL.0000000000007371-10.1212/WNL.000000000 10.1212/WNL.0000000000007371 PMC6511078 30918090

[14] Hadjikhani N, Albrecht DS, Mainero C (2020). Extra-Axial Inflammatory Signal in Parameninges in Migraine with Visual Aura. Ann Neurol.

[15] Merli E, Rustici A, Gramegna LL (2022). Vessel-wall MRI in primary headaches: The role of neurogenic inflammation. Headache J Head Face Pain.

[16] Kim YS, Kim M, Choi SH et al (2019) Altered Vascular Permeability in Migraine-associated Brain Regions: Evaluation with Dynamic Contrast- enhanced MRI. Radiology. 10.1148/radiol.2019182566 10.1148/radiol.2019182566 31264949

[17] Rotstein DL, Aviv RI, Murray BJ (2012). Migraine with aura associated with unilateral cortical increase in vascular permeability. Cephalalgia.

[18] Smith M, Cros D, Sheen V (2002). Hyperperfusion with vasogenic leakage by fMRI in migraine with prolonged aura. Neurology.

[19] Lanfranconi S, Corti S, Bersano A (2009). Aphasic and visual aura with increased vasogenic leakage: an atypical migrainosus status. J Neurol Sci.

[20] Arnold G, Reuter U, Kinze S (1998). Migraine with aura shows gadolinium enhancement which is reversed following prophylactic treatment. Cephalalgia.

[21] Gómez-Choco M, Capurro S, Obach V (2008). Migraine with aura associated with reversible sulcal hyperintensity in FLAIR. Neurology.

[22] Dreier JP, Jurkat-Rott K, Petzold GGCG (2005). Opening of the blood-brain barrier preceding cortical edema in a severe attack of FHM type II. Neurology.

[23] Cha YH, Millett D, Kane M (2007). Adult-onset hemiplegic migraine with cortical enhancement and oedema. Cephalalgia.

[24] Iizuka T, Takahashi Y, Sato M (2012). Neurovascular changes in prolonged migraine aura in FHM with a novel ATP1A2 gene mutation. J Neurol Neurosurg Psychiatry.

[25] Iizuka T, Sakai F, Suzuki K (2006). Implication of augmented vasogenic leakage in the mechanism of persistent aura in sporadic hemiplegic migraine. Cephalalgia.

[26] Pellerin A, Marois C, Mezouar N (2019). Neuronal injuries evidenced by transient cortical magnetic resonance enhancement in hemiplegic migraine: A case report. Cephalalgia.

[27] Pappagallo M, Szabo Z, Esposito G, Lokesh A, Velez L (1999). Imaging neurogenic inflammation in patients with migraine headaches. Neurology.

[28] Schankin CJ, Maniyar FH, Seo Y et al (2016) Ictal lack of binding to brain parenchyma suggests integrity of the blood-brain barrier for 11 C-dihydroergotamine during glyceryl trinitrate-induced migraine. 10.1093/aww112 10.1093/brain/aww096 PMC4939703 27234268

[29] Sianard-Gainko J, Milet J, Ghuysen V, Schoenen J (1994). Increased parasellar activity on Gallium SPECT is not specific for active cluster headache. Cephalalgia.

[30] PubChem Identifier: CID 65430 URL: https://pubchem.ncbi.nlm.nih.gov/compound/Gallium-citrate-ga-67. Accessed 23 Oct 2020.

[31] Farrell BT, Hamilton BE, Dosa E, et al (2013) Using iron oxide nanoparticles to diagnose CNS inflammatory diseases and PCNSL. American Academy of Neurology 10.1212/WNL.0b013e31829bfd8f PMC3770167 23771486

[32] Saleh A, Schroeter M, Jonkmanns C (2004). In vivo MRI of brain inflammation in human ischaemic stroke. Brain.

[33] Neuwelt EA, Várallyay CG, Manninger S (2007). The potential of ferumoxytol nanoparticle magnetic resonance imaging, perfusion, and angiography in central nervous systemt malignancy: a pilot study. Neurosurgery.

[34] Wasserman BA, Smith WI, Trout HH et al (2002) Carotid Artery Atherosclerosis: In Vivo Morphologic Characterization with Gadolinium-enhanced Double-oblique MR Imaging—Initial Results1. 101148/radiol2232010659. 223:566–573 10.1148/radiol.2232010659 11997569

[35] Pannell M, Economopoulos V, Wilson TC (2020). Imaging of translocator protein upregulation is selective for pro-inflammatory polarized astrocytes and microglia. Glia.

[36] Nutma E, Gebro E, Marzin MC et al (2021) Activated microglia do not increase 18 kDa translocator protein (TSPO) expression in the multiple sclerosis brain. 10.1002/glia.24052 10.1002/glia.24052 PMC8453709 34145928

[37] Reuter U, Chiarugi A, Bolay H, Moskowitz MA (2002). Nuclear factor-κB as a molecular target for migraine therapy. Ann Neurol.

[38] Olesen J, Iversen HK, Thomsen LL (1993). Nitric oxide supersensitivity: a possible molecular mechanism of migraine pain. Neuroreport.

[39] Thomsen LL, Kruuse C, Iversen HK, Olesen J (1994). A nitric oxide donor (nitroglycerin) triggers genuine migraine attacks. Eur J Neurol.

[40] Sarchielli P, Floridi A, Mancini ML (2006). NF-κ B activity and iNOS expression in monocytes from internal jugular blood of migraine without aura patients during attacks. © Blackwell Publ Ltd. Cephalalgia.

[41] Sarchielli P, Alberti A, Baldi A (2006). Proinflammatory Cytokines, Adhesion Molecules, and Lymphocyte Integrin Expression in the Internal Jugular Blood of Migraine Patients Without Aura Assessed Ictally. Headache J Head Face Pain.

[42] Markowitz S, Saito K, Moskowitt MA (1987). Neurogenically mediated leakage of plasma protein occurs from blood vessels in dura mater but not brain. J Neurosci.

[43] Lembeck F, Holzer P (1979). Substance P as neurogenic mediator of antidromic vasodilation and neurogenic plasma extravasation. Naunyn Schmiedebergs Arch Pharmacol.

[44] Schwenger N, Dux M, De Col R (2006). Interaction of calcitonin gene-related peptide, nitric oxide and histamine release in neurogenic blood flow and afferent activation in the rat cranial dura mater. Cephalalgia.

[45] Rozniecki JJ, Dimitriadou V, Lambracht-Hall M (1999). Morphological and functional demonstration of rat dura mater mast cell-neuron interactions in vitro and in vivo. Brain Res.

[46] Dimtriadou V, Buzzi MG, Moskowitz MA, Theoharides TC (1991). Trigeminal sensory fiber stimulation induces morphological changes reflecting secretion in rat dura mater mast cells. Neuroscience.

[47] Dux E, Joó F (1982). Effects of Histamine on Brain Capillaries. Exp Brain Res.

[48] Sarker MH, Easton AS, Fraser PA (1998). Regulation of cerebral microvascular permeability by histamine in the anaesthetized rat. J Physiol.

[49] Gursoy-Ozdemir Y, Lo EH, Moskowitz MA (2004). Cortical spreading depression activates and upregulates MMP-9. J Clin Invest.

[50] Gurney KJ, Estrada EY, Rosenberg GA (2006). Blood-brain barrier disruption by stromelysin-1 facilitates neutrophil infiltration in neuroinflammation. Neurobiol Dis.

[51] Bernecker C, Pailer S, Kieslinger P (2011). Increased matrix metalloproteinase activity is associated with migraine and migraine-related metabolic dysfunctions. Eur J Neurol.

[52] Martins-Oliveira A, Speciali JG, Dach F (2009). Different circulating metalloproteinases profiles in women with migraine with and without aura. Clin Chim Acta.

[53] Leira R, Sobrino T, Rodríguez-Yáñez M (2007). MMP-9 Immunoreactivity in Acute Migraine. Headache J Head Face Pain.

[54] Imamura K, Takeshima T, Fusayasu E, Nakashima K (2007). Increased Plasma Matrix Metalloproteinase-9 Levels in Migraineurs. Headache J Head Face Pain.

[55] Ashina M, Tvedskov JF, Lipka K (2009). Matrix metalloproteinases during and outside of migraine attacks without aura. Cephalalgia.

[56] Tsan MF (1985). Mechanism of gallium-67 accumulation in inflammatory lesions. J Nucl Med.

[57] Karatas H, Erdener SE, Gursoy-Ozdemir Y (2011). Spreading Depression Triggers Headache by Activating Neuronal Panx1 Channels. J Acquir Immune Defic Syndr.

[58] Schain AJ, Melo-Carrillo A, Borsook D (2019). Activation of pial and dural macrophages and dendritic cells by CSD (67 chrs). Ann Neurol.

[59] Schain AJ, Melo-Carrillo A, Ashina S (2020). Celecoxib reduces cortical spreading depression-induced macrophage activation and dilatation of dural but not pial arteries in rodents: implications for mechanism of action in terminating migraine attacks. Pain.

[60] Viana M, Linde M, Sances G (2016). Migraine aura symptoms: Duration, succession and temporal relationship to headache. Cephalalgia.

[61] Takizawa T, Qin T, Lopes de Morais A (2020). Non-invasively triggered spreading depolarizations induce a rapid pro-inflammatory response in cerebral cortex. J Cereb Blood Flow Metab.

[62] Yücel M, Kotan D, Gurol ÇG (2016). Serum levels of endocan, claudin-5 and cytokines in migraine. Eur Rev Med Pharmacol Sci.

[63] Zhang X, Burstein R, Levy D (2012). Local action of the proinflammatory cytokines IL-1β and IL-6 on intracranial meningeal nociceptors. Cephalalgia.

[64] Zhang X-C, Kainz V, Burstein R, Levy D (2011). Tumor necrosis factor-alpha induces sensitization of meningeal nociceptors mediated via local COX and p38 MAP kinase actions. Pain.

[65] Bowen EJ, Schmidt TW, Firm CS (2006). Tumor necrosis factor-α stimulation of calcitonin gene-related peptide expression and secretion from rat trigeminal ganglion neurons. J Neurochem.

[66] Herisson F, Frodermann V, Courties G (2018). Direct vascular channels connect skull bone marrow and the brain surface enabling myeloid cell migration. Nat Neurosci.

[67] Grinberg YY, Dibbern ME, Levasseur VA, Kraig RP (2013). Insulin-Like Growth Factor-1 Abrogates Microglial Oxidative Stress and TNF-α Responses to Spreading Depression. J Neurochem.

[68] Jander S, Schroeter M, Peters O (2001). Cortical Spreading Depression Induces Proinflammatory Cytokine Gene Expression in the Rat Brain. J Cereb Blood Flow Metab.

[69] Cagnin A, Gerhard A, Banati RB (2002). In vivo imaging of neuroinflammation. Eur Neuropsychopharmacol.

[70] Sandiego CM, Gallezot JD, Pittman B (2015). Imaging robust microglial activation after lipopolysaccharide administration in humans with PET. Proc Natl Acad Sci.

[71] Takizawa T, Shibata M, Kayama Y et al (2017) High-mobility group box 1 is an important mediator of microglial activation induced by cortical spreading depression. J Cereb Blood Flow Metab. 10.1177/0271678X16647398 10.1177/0271678X16647398 PMC5363469 27142867

[72] Schain AJ, Melo-Carrillo A, Stratton J (2019). CSD-Induced Arterial Dilatation and Plasma Protein Extravasation Are Unaffected by Fremanezumab: Implications for CGRP’s Role in Migraine with Aura. J Neurosci.

[73] Khan S, Amin FM, Christensen CE (2019). Meningeal contribution to migraine pain: A magnetic resonance angiography study. Brain.

